# Cerebrospinal fluid NPTX2/p‐tau ratio as a biomarker for cognitive decline in neurodegenerative diseases

**DOI:** 10.1002/dad2.70391

**Published:** 2026-06-18

**Authors:** Bárbara Fernandes Gomes, Mathias Sauer, Laia Montoliu‐Gaya, Maria Franquesa‐Mullerat, Alexandre Bejanin, Daniel Alcolea, Alberto Lleó, Ignacio Illán‐Gala, Juan Fortea, Kaj Blennow, Henrik Zetterberg, Johanna Nilsson, Olivia Belbin, Nicholas J. Ashton

**Affiliations:** ^1^ Department of Psychiatry and Neurochemistry Institute of Neuroscience and Physiology The Sahlgrenska Academy at the University of Gothenburg Mölndal Sweden; ^2^ Sant Pau Memory Unit, IR SANT PAU Hospital de la Santa Creu i Sant Pau Barcelona Spain; ^3^ Centro de Investigación Biomédica en Red en Enfermedades Neurodegenerativas CIBERNED Madrid Spain; ^4^ Barcelona Down Medical Center Fundació Catalana Síndrome de Down Barcelona Spain; ^5^ Clinical Neurochemistry Laboratory Sahlgrenska University Hospital Mölndal Sweden; ^6^ Paris Brain Institute ICM Pitié‐Salpêtrière Hospital Sorbonne University Paris France; ^7^ Neurodegenerative Disorder Research Center Division of Life Sciences and Medicine and Department of Neurology Institute on Aging and Brain Disorders University of Science and Technology of China and First Affiliated Hospital of USTC Hefei P.R. China; ^8^ Department of Neurodegenerative Disease UCL Institute of Neurology London UK; ^9^ UK Dementia Research Institute at UCL London UK; ^10^ Hong Kong Center for Neurodegenerative Diseases Clear Water Bay Hong Kong China; ^11^ Wisconsin Alzheimer's Disease Research Center University of Wisconsin School of Medicine and Public Health University of Wisconsin–Madison Madison Wisconsin USA; ^12^ Banner Alzheimer's Institute and University of Arizona Phoenix Arizona USA; ^13^ Banner Sun Health Research Institute Sun City Arizona USA

**Keywords:** Alzheimer's disease, cerebrospinal fluid, cognitive decline, dementia with Lewy bodies, frontotemporal lobar degeneration, neuronal pentraxin 2, phosphorylated tau, single molecule array, synaptic protein ratio

## Abstract

**INTRODUCTION:**

Neuronal pentraxin 2 (NPTX2) and its use as a ratio with other synaptic proteins has emerged as a prognostic cerebrospinal fluid (CSF) biomarker across neurodegenerative diseases.

**METHODS:**

Using a single molecule array (Simoa) method, CSF NPTX2 was measured in 688 individuals from the Sant Pau Initiative on Neurodegeneration, including Alzheimer's disease (AD), dementia with Lewy bodies (DLB), frontotemporal lobar degeneration–related disorders (FTLDrs), and cognitively unimpaired (CU) participants. NPTX2/phosphorylated‐tau (p‐tau)181 performance was compared to standalone NPTX2 and p‐tau181.

**RESULTS:**

The NPTX2/p‐tau ratio enhanced diagnostic performance of standalone NPTX2 and p‐tau, particularly for DLB and FTLDrs (area under the curve [AUC]_NPTX2/p‐tau _= 0.78–0.79 vs. AUC_NPTX2 _= 0.63–0.70 and AUC_p‐tau _= 0.59–0.75), and was more strongly associated with cognition. It also better predicted progression to dementia across the cohort (hazard ratio [HR] = 1.63), especially in AD (HR = 1.84) and DLB (HR = 1.50).

**DISCUSSION:**

NPTX2/p‐tau may improve prognostic assessments in patients with cognitive impairment, outperforming standalone biomarkers.

## Highlights

- Neuronal pentraxin 2 (NPTX2) is a robust synaptic biomarker in neurodegenerative diseases.
- NPTX2/phosphorylated‐tau_1_ (p‐tau) enhances diagnostic accuracy of the standalone markers.
- NPTX2/p‐tau improves associations with cognition and cognitive decline.
- The ratio is the strongest predictor of dementia, compared to NPTX2 and p‐tau alone.
- The NPTX2/p‐tau ratio may improve prognostic assessments in clinical routine.

## 1 INTRODUCTION

Neuronal pentraxin 2 (NPTX2), first described in 1995,^1^ is part of the pentraxin family, along with neuronal pentraxin 1 and neuronal pentraxin receptor, with which it shares homology.^2^ NPTX2 is expressed in both several peripheral tissues^3^ and in the brain, where it localizes to presynaptic terminals of excitatory synapses and contributes to synaptic plasticity, pruning, and trans‐synaptic signaling. It is released into the synaptic cleft during high neuronal activity and promotes synaptogenesis by acting on the postsynaptic terminal.^2^, ^4^, ^5^

Given its involvement in synaptic signaling, NPTX2 has been studied as a biomarker for Alzheimer's disease (AD), in which synaptic dysfunction is considered an early sign of neurodegeneration.^6^, ^7^ Reduced NPTX2 levels have been reported in cerebrospinal fluid (CSF) from patients with mild cognitive impairment (MCI) and AD, and has been reported to correlate with amyloid beta (Aβ)42 in CSF, cortical thickness, and cognitive impairment.^8^, ^9^, ^10^, ^11^ Additionally, NPTX2 was shown to be decreased in the *post mortem* cortex of AD patients,^11^ supporting its role as a marker of synaptic dysfunction and loss.

**RESEARCH IN CONTEXT**

1. **Systematic review**: The authors searched literature using PubMed, as well as recent conference abstracts, to identify studies examining cerebrospinal fluid (CSF) neuronal pentraxin 2 (NPTX2) and its relationship to cognition, neurodegeneration, and tau markers in Alzheimer's disease (AD), dementia with Lewy bodies (DLB), and frontotemporal lobar degeneration–related disorders (FTLDrs). Prior studies consistently report lower levels of NPTX2 in these dementias. However, few studies have evaluated NPTX2 in large, clinical diverse cohorts or assessed its performance in combination with phosphorylated‐tau (p‐tau)181.
2. **Interpretation**: Our findings show that the NPTX2/p‐tau ratio improves group separation and association with cognition and neuropsychiatric symptoms, relative to each standalone biomarker, particularly in DLB and FTLDrs.
3. **Future directions**: Future studies should evaluate NPTX2 and the NPTX2/p‐tau ratio in pathology‐confirmed cohorts, as well as compare them to additional synaptic markers and other phosphorylated tau species (e.g., p‐tau217).

However, changes in synapse density are not exclusive to AD. Parkinson's disease (PD) and dementia with Lewy bodies (DLB), both defined by the presence of α‐synuclein (αSyn) inclusions,^12^ are highly associated with synaptic dysfunction and loss of integrity.^13^, ^14^ Similarly, synaptic loss has also been observed in frontotemporal lobar degeneration (FTLD), a group of diseases characterized by deficits in behavior, language, and executive function.^15^ Reduced NPTX2 levels have been reported in the CSF of patients with PD, DLB, and the behavioral variant of frontotemporal dementia (bvFTD), the most prevalent syndrome in the FTLD spectrum.^9^, ^10^, ^16^, ^17^, ^18^ Furthermore, ratios between NPTX2 and other synaptic biomarkers, such as synaptosomal‐associated protein, 25kDa (SNAP‐25) and 14‐3‐3 zeta/delta, have also been shown to improve discrimination among neurodegenerative diseases and increase association with cognitive decline.^9^

Taken together, this suggests NPTX2 is not a disease‐specific biomarker, but instead a valuable marker for synaptic dysfunction and tracking disease progression across neurodegenerative disorders. However, immunoassays suitable for high clinical throughput for measuring NPTX2 have been limited, which was recently addressed by the development of single molecule array (Simoa).^19^ Furthermore, few studies have assessed the use of ratios with synaptic proteins, with the majority investigating their performance in the AD continuum and not in non‐AD diseases.^9^, ^20^

Here, we analyzed CSF NPTX2 in patients with various neurodegenerative diseases, including DLB, FTLD‐related syndromes (FTLDrs), and AD, using a novel Simoa NPTX2 assay and assessed the diagnostic utility of the ratio of NPTX2 over phosphorylated‐tau (p‐tau)181 and its association with cognition and cognitive decline.

## 2 MATERIALS AND METHODS

### 2.1 Clinical cohort

Participants in this study were from the Sant Pau Initiative on Neurodegeneration (SPIN, Table 1) and evaluated at the Sant Pau Memory Unit (Barcelona, Spain).^21^ The cohort included patients with AD (prodromal or AD dementia, *n* = 287), DLB (*n* = 118), and FTLDrs (*n* = 121), comprising bvFTD, semantic variant primary progressive aphasia (svPPA), non‐fluent variant primary progressive aphasia (nfvPPA), corticobasal syndrome (CBS) and progressive supranuclear palsy (PSP). Cognitively unimpaired (CU, *n* = 159) individuals were required to have normal neurological and cognitive assessment, and normal CSF AD biomarkers.

**TABLE 1:** Baseline characteristics of the cohort.

	CU *N* = 159	AD *N* = 288	DLB *N* = 118	FTLDrs *N* = 121	*p* value
**Age (y)**	56 (49, 64)	75 (70, 78)	77 (72, 80)	72 (68, 78)	<0.001
**Sex**					<0.001
Female	88 (55%)	184 (64%)	55 (47%)	53 (44%)	
Male	71 (45%)	103 (36%)	63 (53%)	68 (56%)	
**Education (y)**	17 (13, 20)	9 (8, 13)	8 (7, 12)	12 (9, 16)	<0.001
**MMSE score**	30 (29, 30)	24 (22, 26)	25 (22, 27)	26 (21, 28)	<0.001
**GDS**					
1	157 (99%)	0 (0%)	0 (0%)	0 (0%)	
2	1 (0.6%)	0 (0%)	0 (0%)	0 (0%)	
3	0 (0%)	173 (60%)	61 (52%)	65 (54%)	
4	0 (0%)	110 (38%)	43 (37%)	40 (33%)	
5	0 (0%)	3 (1.0%)	10 (8.6%)	13 (11%)	
6	0 (0%)	1 (0.3%)	2 (1.7%)	2 (1.6%)	
7	0 (0%)	0 (0%)	0 (0%)	0 (0%)	
**Aβ42 (pg/mL)**	1154 (936, 1455)	534 (423, 634)	715 (536, 1020)	892 (622, 1171)	<0.001
**Aβ40 (pg/mL)**	11,483 (9340, 14,044)	12,518 (10,065, 14,712)	10,711 (9067, 14,165)	9581 (7704, 12,534)	<0.001
**t‐tau (pg/mL)**	251 (190, 317)	664 (504, 906)	371 (264, 531)	331 (238, 460)	<0.001
**CSF NPTX2 (pg/mL)**	29,173 (18,667, 46,577)	23,364 (15,227, 35,827)	20,279 (12,659, 40,470)	19,271 (12,138, 28,073)	<0.001
**CSF p‐tau (pg/mL)**	33.1 (26.1, 44.2)	105.6 (78.9, 143.0)	51.2 (35.4, 79.1)	39.3 (29.7, 51.4)	<0.001
**NPTX2/p‐tau ratio**	816 (601, 1261)	209 (131, 328)	389 (203, 666)	472 (295, 716)	<0.001

*Notes*: Values are median (IQR) or n (%); *p* values were calculated using Kruskal–Wallis rank‐sum test for continuous variables and Pearson chi‐squared test for categorical variables.

Abbreviations: Aβ, amyloid beta; AD, Alzheimer's disease; CSF, cerebrospinal fluid; CU, cognitively unimpaired; FTLDrs, frontotemporal lobar degeneration–related disorders; GDS, Global Deterioration Scale; IQR, interquartile range; MMSE, Mini‐Mental State Examination; NPTX2, neuronal pentraxin 2; p‐tau, phosphorylated tau; t‐tau, total tau.

All participants underwent neurological and neuropsychological evaluations. A subset (*n* = 391, 57%) had longitudinal follow‐up for an average of 3.2 years. Core AD biomarkers (Aβ42 or Aβ42/Aβ40 ratio, p‐tau, and total tau [t‐tau]) were assessed using validated local cutoffs with high diagnostic accuracy.^22^ Further information can be found in Text S1 in supporting information.

### 2.2 CSF collection and biomarker assessment

CSF samples were collected as previously described.^21^ Samples were stored at −80°C and were not thawed prior to analysis. Commercially available immunoassays were used on the LUMIPULSE G600II platform to determine levels of CSF Aβ42, Aβ40, t‐tau, and p‐tau 181 (Lumipulse G assays β‐Amyloid 1‐40 and 1‐42, t‐tau, p‐tau_181_ from Fujirebio). Samples were blinded for clinical diagnosis and randomized before NPTX2 analysis.

### 2.3 NPTX2 measurements

CSF NPTX2 was quantified at the University of Gothenburg on a Simoa HD‐X instrument (Quanterix), using a recently developed in‐house assay.^19^ Monoclonal antibodies were used for capture (ab191563, Abcam) and detector (ab277533, Abcam). Full‐length recombinant NPTX2 was used as calibrator (7816‐NP‐050, Biotechne). Samples were run in singlicate with internal quality controls. Full assay details are provided in Text S2 in supporting information.

### 2.4 Magnetic resonance imaging acquisition and analysis

3D T1‐weighted magnetic resonance imaging (MRI) was acquired on 3T scanners at two sites. Processing followed CAT12/SPM12 pipelines, with gray‐matter maps smoothed for voxel‐wise analysis. After quality control, 249 scans were included. Full imaging parameters and preprocessing details are provided in Text S3 and Table S1 in supporting information.

### 2.5 Statistical analysis

All analyses were performed in R (v4.4.0). Demographic differences were tested using analysis of variance or chi‐squared tests. Group comparisons for NPTX2, p‐tau, and NPTX2/p‐tau used log‐transformed linear models with false discovery rate correction. Receiver operating characteristic analyses and DeLong tests evaluated diagnostic performance. Associations with clinical and cognitive measures were assessed using linear, mixed‐effect, or proportional‐odds models, and progression to dementia was analyzed with Cox regression. Voxel‐wise neuroimaging analyses were performed within a gray‐matter mask, adjusted for intracranial volume, and multiple comparisons were corrected using family‐wise error. All models were adjusted for age, sex, and education. Full statistical procedures are provided in Text S4 in supporting information.

## 3 RESULTS

### 3.1 Lower NPTX2/p‐tau levels across neurodegenerative diseases

**FIGURE 1:**
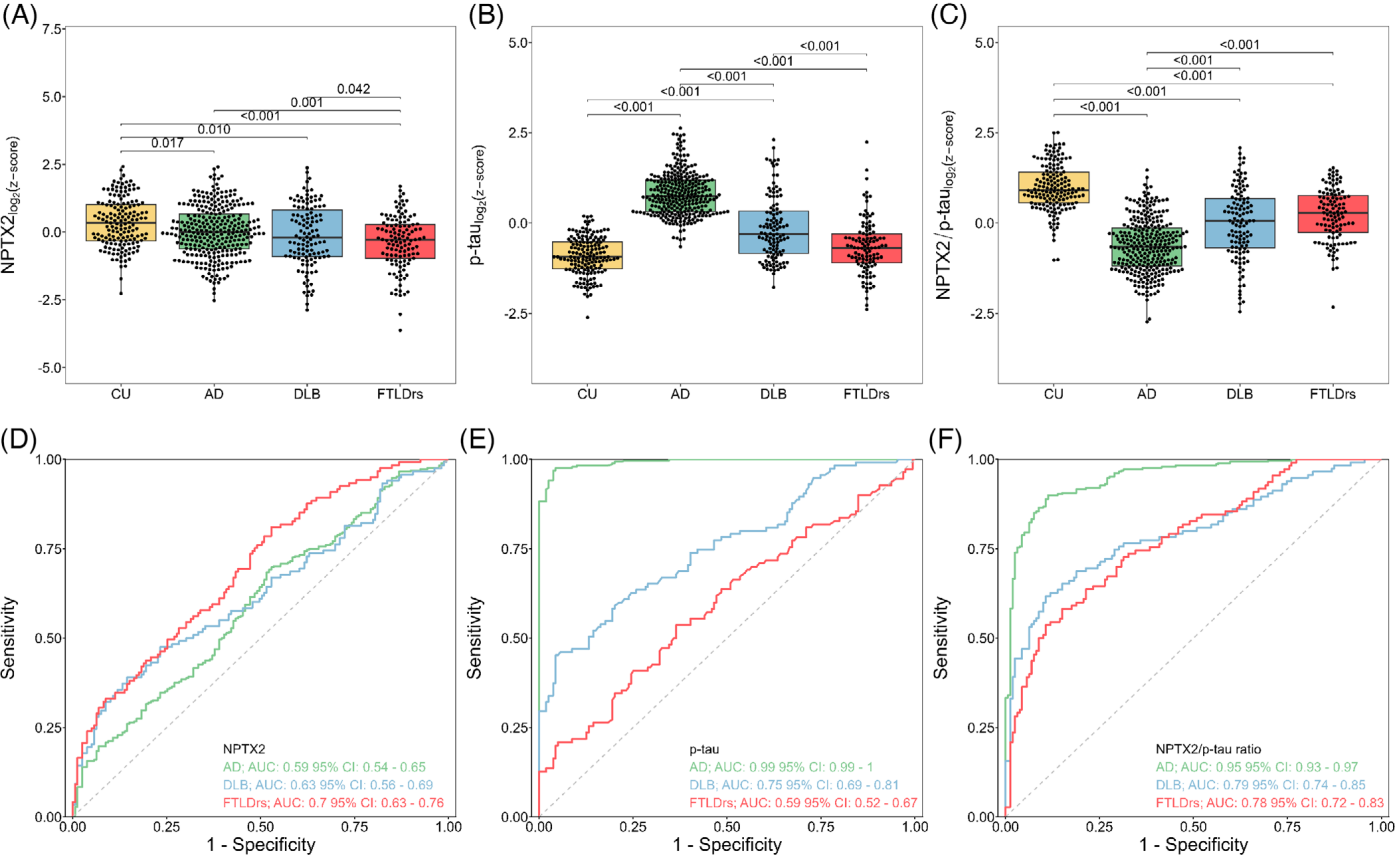
Biomarker group‐wise comparison. A, CSF NPTX2 levels in CU (*n* = 159), AD (*n* = 287), DLB (*n* = 118), and FTLDrs (*n* = 121). B, CSF p‐tau in CU (*n* = 159), AD (*n* = 287), DLB (*n* = 115), and FTLDrs (*n* = 110). C, NPTX2/p‐tau ratio in CU (*n* = 159), AD (*n* = 287), DLB (*n* = 115), and FTLDrs (*n* = 110). D, ROC curves for NPTX2 across the diagnostic groups compared to CU individuals. E, ROC for p‐tau across the diagnostic groups compared to CU individuals. F, ROC curves for NPTX2/p‐tau across the diagnostic groups compared to CU individuals. *p* values in boxplots were obtained from linear models adjusted for age, sex, and education. Horizontal line within boxplots denotes median, top and bottom of boxplots represent the 25th and 75th percentiles, respectively, and whiskers extend to 1.5x interquartile range. AD, Alzheimer's disease; AUC, area under the curve; CI, confidence interval; CSF, cerebrospinal fluid; CU, cognitively unimpaired; DLB, dementia with Lewy bodies; FTLDrs, frontotemporal lobar degeneration–related disorders; NPTX2, neuronal pentraxin 2; p‐tau, phosphorylated tau; ROC, receiver operating characteristic.

CSF NPTX2 levels across the neurodegenerative diseases in the SPIN cohort can be found in Figure 1A. NPTX2 levels were lower in patients with AD (β = −0.33, standard error [SE] = 0.13, *P *= 0.011), DLB (β = −0.43, SE = 0.15, *P *< 0.01), and FTLDrs (β = −0.70, SE = 0.14, *P *< 0.001), compared to CU individuals. The lowest levels were found in FTLDrs, which were lower than in AD (β = −0.37, SE = 0.11, *P *< 0.001) and DLB (β = −0.32, SE = 0.15, *P *= 0.03) groups. As a key inclusion criterion for individuals in the AD group, CSF p‐tau was increased in AD compared to CU (β = 1.45, SE = 0.09, *P *< 0.001). Additionally, p‐tau was also increased in DLB compared to the CU group (β = 0.47, SE = 0.11, *P *< 0.001), while no significant changes were observed for the FTLDrs group (Figure 1B).

NPTX2 was then used as a ratio with p‐tau (NPTX2/p‐tau), thereby controlling for varying levels of p‐tau across the diagnostic groups (Figure 1C). NPTX2/p‐tau showed a greater statistical separation between diagnostic groups than NPTX2 alone, with a decrease in AD (β = −1.43, SE = 0.11, *P *< 0.001), DLB (β = −0.74, SE = 0.13, *P *< 0.001), and FTLDrs (β = −0.57, SE = 0.12, *P *< 0.001) groups, compared to CU individuals. Additionally, the ratio improved the separation between DLB and CU (Figure S1A–C in supporting information), as well as between the FTLDrs and CU (Figure S1D–F) groups, compared to p‐tau. NPTX2/p‐tau could also better differentiate AD from DLB (β = −0.68, SE = 0.10, *P *< 0.001) and FTLDrs (β = −0.87, SE = 0.09, *P *< 0.001), compared to NPTX2. NPTX2, p‐tau, and NPTX2/p‐tau levels in FTLDrs subgroups are shown in Figure S2 in supporting information.

To assess diagnostic performance, area under the curve (AUC) values for NPTX2/p‐tau and the standalone markers were compared (Figure 1D‐F). NPTX2 alone exhibited poor discriminatory power for AD (AUC = 0.59, 95% confidence interval [CI]: 0.54–0.65), and moderate for DLB and FTLDrs (AUC = 0.63, 95% CI: 0.56–0.69 and AUC = 0.70, 95% CI: 0.64–0.76, respectively; Figure 1D). Conversely, as a defining feature, p‐tau levels were the best at differentiating AD from CU individuals (AUC = 0.99, 95% CI: 0.99–1.00), while exhibiting moderate diagnostic accuracy for DLB (AUC = 0.75, 95% CI: 0.69–0.81) and poor discriminatory power for FTLDrs individuals (AUC = 0.59, 95% CI: 0.52–0.67; Figure 1E).

NPTX2/p‐tau vastly improved diagnostic performance for the AD (AUC = 0.95, 95% CI: 0.93–0.97), DLB (AUC = 0.79, 95% CI: 0.74–0.85), and FTLDrs (AUC = 0.78, 95% CI: 0.72–0.83) groups, compared to NPTX2 (DeLong, *P *< 0.001 for all groups; Figure 1F). Furthermore, NPTX2/p‐tau also improved the discrimination between FTLDrs and CU, compared to p‐tau (DeLong, *P *< 0.001). Despite the difference between the DLB and CU groups being larger with NPTX2/p‐tau, compared to p‐tau, there was no difference in discriminatory power comparing the AUC values between the two markers (DeLong, *P *> 0.05). All comparisons are shown in Table S2 in supporting information.

### 3.2 Association of NPTX2/p‐tau with cognitive decline and its progression

**FIGURE 2:**
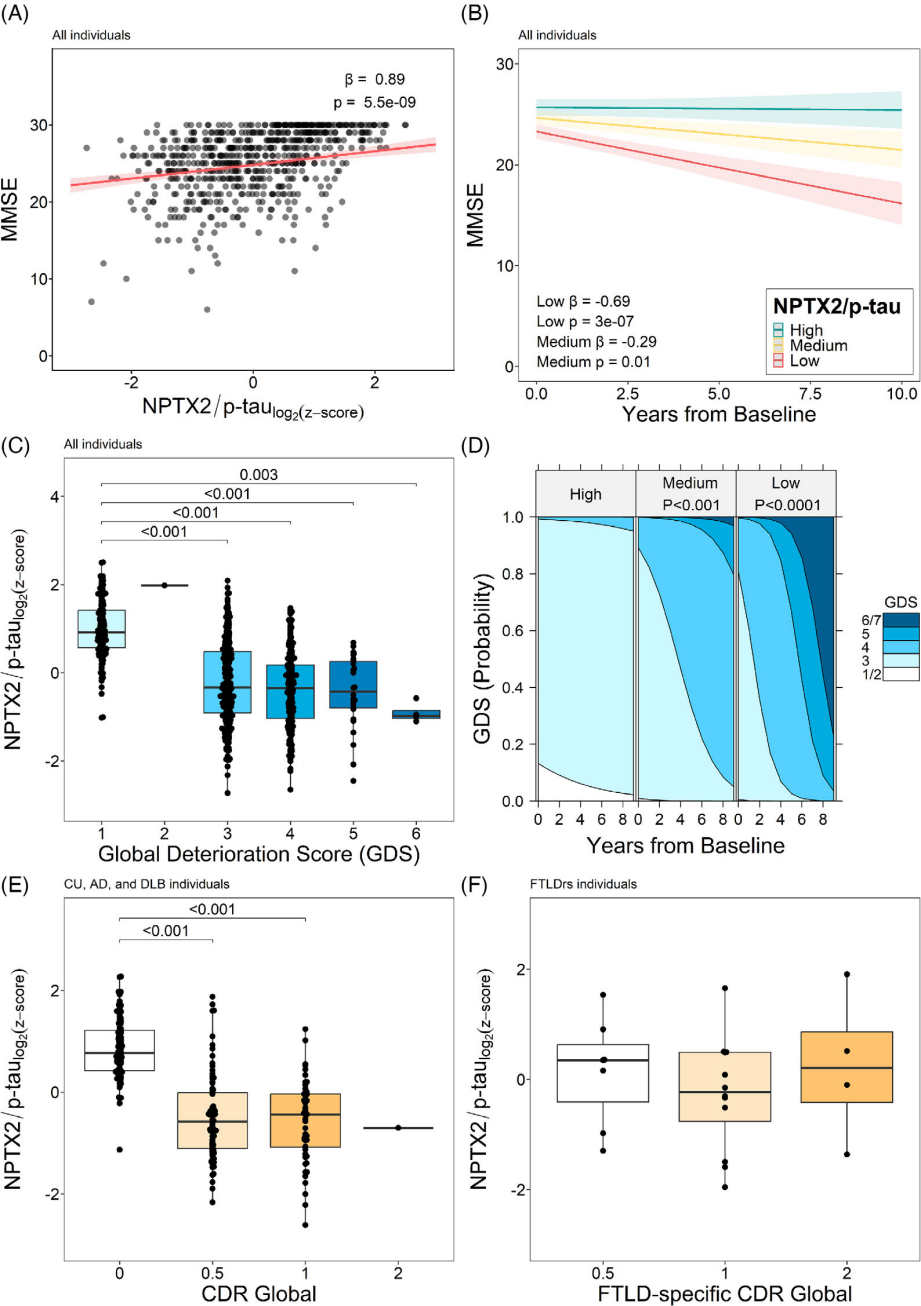
Cross‐sectional and longitudinal NPTX2/p‐tau ratio association with MMSE, GDS, and CDR for all individuals. A, Association of NPTX2/p‐tau with MMSE, at baseline (*n* = 674). B, Longitudinal trajectory of MMSE according to NPTX2/p‐tau levels (1288 data points from 371 individuals followed up for an average of 3.18 years [SD of 2.06]). C, NPTX2/p‐tau levels across the GDS, at baseline (*n* = 681). D, Longitudinal probability of a given GDS according to NPTX2/p‐tau levels (tertiles; 1287 data points from 371 individuals followed up for an average of 3.18 years [SD of 2.06]). E, NPTX2/p‐tau levels across global CDR in CU, AD, and DLB individuals (*n* = 270), at baseline. F, NPTX2/p‐tau levels across FTLD‐specific global CDR in FTLDrs individuals (*n* = 24), at baseline. All analyses were obtained from linear regression, linear mixed models (random slopes and intercept), or ordered logistic regression, with age, sex, and education as covariates and with false discovery rate correction performed for multiple comparisons in (C), (E), and (F). NPTX2/p‐tau ratio was log‐transformed to aid data visualization and estimate interpretation. All estimates and *p* values in (B) and (D) are compared to NPTX2/p‐tau‐High. AD, Alzheimer's disease; CDR, Clinical Dementia Rating; DLB, dementia with Lewy bodies; FTLDrs, frontotemporal lobar degeneration–related disorders; GDS, Global Deterioration Scale; MMSE, Mini‐Mental State Examination; NPTX2, neuronal pentraxin 2; p‐tau, phosphorylated tau; SD, standard deviation.

We observed that NPTX2/p‐tau correlated with the Mini‐Mental State Examination (MMSE) score at baseline in the whole cohort (β = 0.89, SE = 0.15, *P *< 0.0001; Figure 2A), and in the AD (β = 0.43, SE = 0.21, *P *< 0.05) and DLB (β = 1.13, SE = 0.39, *P *< 0.01) groups (Figure S3A–C in supporting information). At baseline, p‐tau had a weaker association with MMSE (β = −0.62, SE = 0.15, *P *< 0.0001) compared to NPTX2/p‐tau (Figure S4A in supporting information) and, unlike NPTX2/p‐tau, only had a significant association within DLB (β = −1.14, SE = 0.38, *P *< 0.01; Figure S3D‐F). NPTX2 only correlated with MMSE scores in the whole cohort (β = 0.46, SE = 0.14, *P *< 0.01; Figure S5A in supporting information, Figure S3G‐I).

Associations between baseline NPTX2/p‐tau and longitudinal MMSE were also assessed (Figure 2B). Patients with low and medium levels of NPTX2/p‐tau at baseline had a faster decline in MMSE, compared to those with higher levels of NPTX2/p‐tau (β = −0.69, SE = 0.13, *P *< 0.0001 and β = −0.29, SE = 0.12, *P *= 0.01, respectively). Similarly, patients with high and medium levels of p‐tau had a faster decline in MMSE than those with lower levels (β = −0.78, SE = 0.13, *P *< 0.0001 and β = −0.24, SE = 0.18, *P *< 0.05; Figure S4B). NPTX2 was not associated with MMSE decline (Figure S5B). No biomarker had a longitudinal association with MMSE decline in a specific diagnostic group (data not shown).

NPTX2/p‐tau was evaluated in relation to the Global Deterioration Scale (GDS), a 7‐stage measure of global and functional decline. NPTX2/p‐tau levels were lower across the GDS levels 3 (β = −1.01, SE = 0.11, *P *< 0.0001), 4 (β = −1.12, SE = 0.12, *P *< 0.0001), 5 (β = −1.23, SE = 0.20, *P *< 0.0001), and 6 (β = −1.62, SE = 0.49, *P *< 0.01), compared to GDS 1 (Figure 2C), to a greater extent than p‐tau (GDS level 3 [β = 0.86, SE = 0.11, *P *< 0.0001], 4 [β = 0.89, SE = 0.13, *P *< 0.0001], 5 [β = 0.55, SE = 0.20, *P *< 0.05], and 6 [β = 0.13, SE = 0.50, *P = *0.78], compared to GDS 1; Figure S4C), at baseline. Conversely, NPTX2 levels were consistently lower as GDS scores increased, outperforming p‐tau and NPTX2/p‐tau to discriminate between GDS scores (Figure S5C). The GDS was also assessed longitudinally (Figure 2D,  Figure S4D and S5D), using an ordered logistic mixed‐effects model to estimate the probability of a given GDS score over time across biomarker strata. We observed increasing probability of higher GDS score in both low NPTX2/p‐tau levels over time (odds ratio [OR] = 2.21, 95% CI: 1.77–2.78, *P *< 0.0001), and medium NPTX2/p‐tau levels (OR = 1.41, 95% CI: 1.15–1.74, *P *< 0.001) in all individuals (Figure 2D), and specifically in the DLB group (low levels: OR = 1.82, 95% CI: 1.31–2.51, *P *< 0.001; medium levels: OR = 1.40, 95% CI: 1.06–1.84, *P *< 0.05), compared to higher NPTX2/p‐tau levels (Figure S6 in supporting information). Conversely, p‐tau was associated with the longitudinal changes in GDS for high levels (OR = 1.66, 95% CI: 1.33–2.08, *P *< 0.0001) in all individuals (Figure S4D). Lower levels of NPTX2 were associated with GDS score progression (OR = 1.61, 95% CI: 1.31–1.98, *P *< 0.0001; Figure S5D). Overall, the association between the NPTX2/p‐tau (OR = 2.21) ratio and GDS progression was stronger than that observed for both NPTX2 (OR = 1.61) or p‐tau (OR = 1.66), especially in patients with DLB.

Next, we evaluated global Clinical Dementia Rating (CDR) and FTLD‐CDR to understand how NPTX2/p‐tau levels might vary across different cognition states. We found that NPTX2/p‐tau levels in the CU, AD, and DLB group were lower in CDR 0.5 (β = 1.23, SE = 0.15, *P *< 0.001) and CDR 1 (β = 1.31, SE = 0.17, *P *< 0.001), compared to CDR 0 (Figure 2E). Similarly, p‐tau was increased at CDR 0.5 (β = 1.05, SE = 0.14, *P *< 0.001) and CDR 1 (β = 1.15, SE = 0.16, *P *< 0.001), compared to CDR 0 (Figure S4E; NPTX2 levels showed a small decrease across CDR levels (CDR 0.5 β = −0.62, SE = 0.20, *P *< 0.01; CDR 1 β = −0.59, SE = 0.22, *P *< 0.01 compared to CDR 0; Figure S5E). No significant differences were found in patients with FTLDrs (Figure 2F, Figure S4–S5F). Together, this suggests that NPTX2/p‐tau might better reflect changes in CDR compared to p‐tau or NPTX2.

**FIGURE 3:**
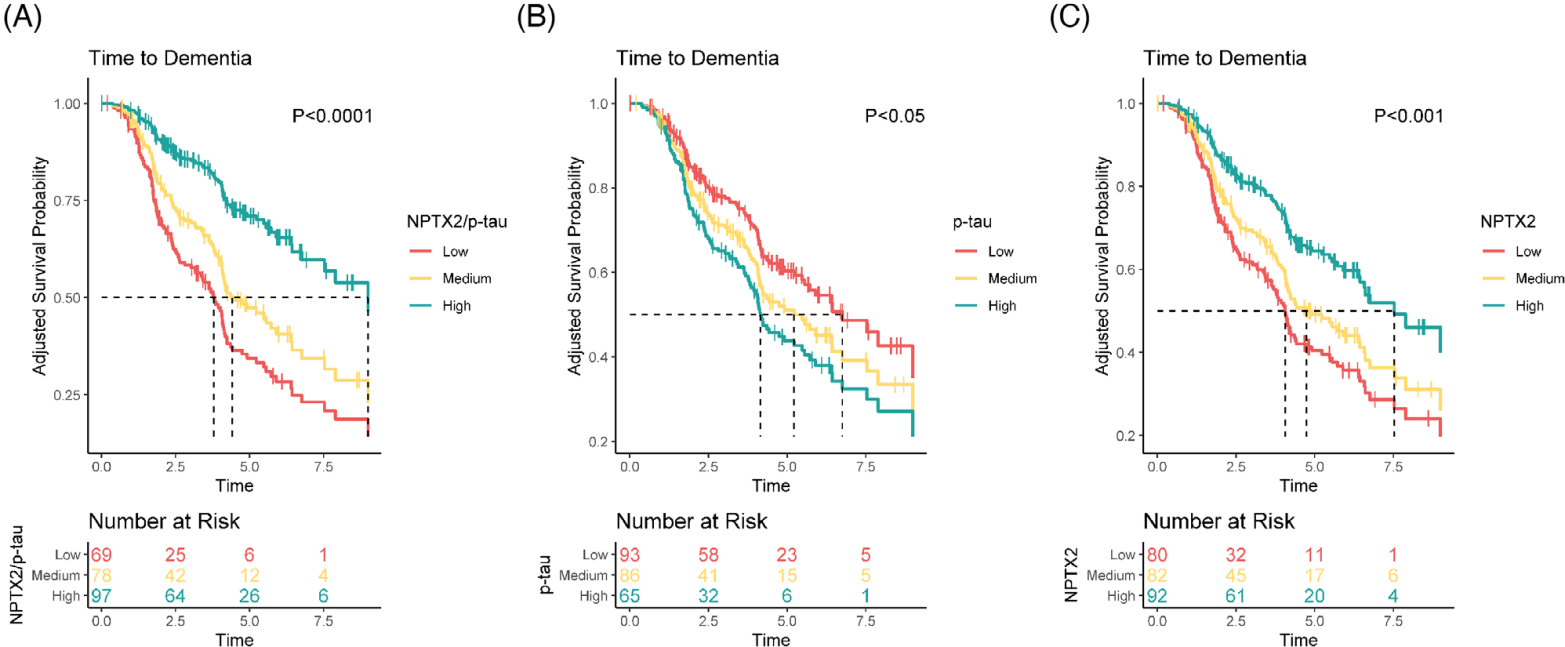
Adjusted Kaplan–Meier curves depicting progression of individuals with normal cognition or MCI to dementia; (A) NPTX2 (*n* = 254), (B) p‐tau (*n* = 246), (C) NPTX2/p‐tau (*n* = 246). Statistical models were performed with continuous variables, while tertiles were used for visualization purposes. *p* value and curves reported in plots reflect Cox regression analysis adjusted for age, sex, and education. MCI, mild cognitive impairment; NPTX2, neuronal pentraxin 2; p‐tau, phosphorylated tau.

Based on the follow‐up data, we evaluated the likelihood of individuals with normal cognition or MCI (GDS < 4) progressing to dementia (GDS ≥ 4) according to the NPTX2/p‐tau, using Cox regression analysis (Figure 3); 45% of these individuals converted to dementia, with an average time of 2.8 years (standard deviation = 1.8).

NPTX2/p‐tau was the strongest predictor of progression to dementia within 10 years in the full cohort (HR = 1.63, 95% CI: 1.35–1.96, *P *< 0.0001), especially in AD (HR = 1.84, 95% CI: 1.31–2.59, *P *< 0.001), and to a lesser extent in DLB (HR = 1.50, 95% CI: 1.10–2.05, *P *< 0.05), but not in FTLDrs (Figure S7A–C in supporting information). P‐tau had the weakest performance when predicting conversion to dementia (HR = 1.35, 95% CI: 0.57–0.97, *P *< 0.05) and could not predict conversion in any specific diagnostic group. Lower NPTX2 levels also increased dementia risk (HR = 1.41, 95% CI: 1.16–1.70, *P *< 0.001; Figure 3A) in all individuals, as well as in those with AD (HR = 1.41, 95% CI: 1.08–1.83, *P *< 0.01) and DLB (HR = 1.49, 95% CI: 1.06–2.09, *P *< 0.05), but not in those with FTLDrs (Figure S7D–F).

Last, we assessed the relationship between NPTX2/p‐tau and cognitive domains, assessed through different cognitive tests (Table 2). Overall, NPTX2/p‐tau was highly associated with all cognitive domains, as was p‐tau. NPTX2 correlated with all domains except visual memory and visuospatial skills, with weaker association than NPTX2/p‐tau and p‐tau. P‐tau was more associated with episodic memory (Free and Cued Selective Reminding Test, β = −0.49, SE = 0.05, *P *< 0.0001), visual memory (Rey Complex Figure Recall, β = −0.31, SE = 0.07, *P *< 0.0001), and visuospatial skills (Visual Object Space and Perception Battery, β = −0.32, SE = 0.07, *P *< 0.0001), while NPTX2/tau better mirrored changes in all other cognitive domains, including attention/executive function (phonemic fluency, β = 0.16, SE = 0.03, *P *< 0.0001; Trail‐Making Test Part A, β = 0.28, SE = 0.04, *P *< 0.0001; Trail‐Making Test Part B, β = 0.27, SE = 0.04, *P *< 0.0001), language (semantic fluency, β = 0.19, SE = 0.03, *P *< 0.0001), and visuospatial skills (geometric figures copy subtest of Consortium to Establish a Registry for Alzheimer's Disease, β = 0.18, SE = 0.06, *P *< 0.01). In AD, NPTX2/p‐tau and p‐tau showed similar association with memory (β = 0.23, SE = 0.12, and β = −0.23, SE = 0.11, *P *< 0.05, respectively) (Table S3 in supporting information). NPTX2/p‐tau also better reflected attention/executive function and language in both AD and DLB groups (Table S3). Visuospatial skills were consistently associated with NPTX2/p‐tau, including in those with DLB (β *= *1.05, SE = 0.36, *P *< 0.01; Table S3). P‐tau also showed association with visuospatial skills both within the DLB (β = −1.03, SE = 0.33, *P *< 0.01) and AD (β = −0.22, SE = 0.11, *P *< 0.05) groups (Table S3).

**TABLE 2:** Association of NPTX2/p‐tau and different cognitive domains compared to NPTX2 and p‐tau in all individuals.

		NPTX2/p‐tau		p‐tau		NPTX2	
Domain	**Test**	**β (SE)**	***p***	**β (SE)**	***p***	**β (SE)**	***p***
**Episodic memory**	^23^FCSRT^1^	0.45 (0.05)	<0.0001^a^	−0.49 (0.05)	<0.0001^a^	0.11 (0.05)	<0.05
	^24^CERAD^1^ Word List	0.30 (0.05)	<0.0001	−0.29 (0.05)	<0.0001	0.11 (0.05)	ns
**Visual memory**	^23^Complex Rey Figure Recall^1^	0.30 (0.07)	<0.0001	−0.31 (0.07)	<0.0001	0.11 (0.06)	ns
**Attention/executive function**	^25^Phonemic Fluency^2^	0.16 (0.03)	<0.0001^b^	−0.11 (0.04)	<0.01	0.07 (0.03)	<0.05
	^26^TMT‐A^1^	0.28 (0.04)	<0.0001^a^ ^,^ ^b^	−0.22 (0.05)	<0.0001^b^	0.12 (0.04)	<0.01
	^26^TMT‐B^1^	0.27 (0.04)	<0.0001	−0.21 (0.05)	<0.0001	0.12 (0.04)	<0.01
**Language**	^25^Semantic fluency^2^	0.19 (0.03)	<0.0001^a^ ^,^ ^b^	−0.13 (0.03)	<0.0001	0.09 (0.02)	<0.001^a^
**Visuospatial skills**	^24^Geometric figures copy subtest of CERAD^1^	0.18 (0.06)	<0.01	−0.15 (0.07)	<0.05	0.07 (0.05)	ns
	^27^VOSP^1^	0.25 (0.06)	<0.001^a^	−0.32 (0.07)	<0.0001^a^ ^,^ ^b^	0.05 (0.06)	ns
	^28^Poppelreuter total^2^	0.02 (0.01)	<0.05	−0.02 (0.01)	<0.05^b^	0.01 (0.01)	ns

Abbreviations: AD, Alzheimer's disease; CERAD, Consortium to Establish a Registry for Alzheimer's Disease; DLB, dementia with Lewy bodies; FCSRT, Free and Cued Selective Reminding Test; FTLDrs, frontotemporal lobar degeneration–related disorders; NPTX2, neuronal pentraxin 2; p‐tau, phosphorylated tau; SE, standard error; TMT‐A, Trail‐Making Test Part A; TMT‐B, Trail‐Making Test Part B; VOSP, Visual Object Space and Perception.

^1^ Linear model on standardized outcome (TMT A/B log‐transformed).

^2^ Quasi‐Poisson generalized linear model for discrete or over‐dispersed counts.

^a^ Significant in AD.

^b^ Significant in DLB.

^c^ Significant in FTLDrs.

### 3.3 Association of NPTX2/p‐tau with psychiatric features

Given the prevalence of psychiatric symptoms in AD, DLB, and FTLDrs, we examined Neuropsychiatric Inventory (NPI) scores in relation to NPTX2/p‐tau and compared these associations with NPTX2 and p‐tau. Results for all NPI subscores are shown in Table 3.

**TABLE 3:** Association of NPTX2/p‐tau and different NPI subscores compared to NPTX2 and p‐tau in all individuals.

	NPTX2/p‐tau		p‐tau		NPTX2	
NPI	β (SE)	*p*	β (SE)	*p*	β (SE)	*p*
Delusions	−0.46 (0.32)	ns^c^	0.35 (0.31)	ns	−0.15 (0.26)	ns^c^
Hallucinations	−0.08 (0.45)	ns^b^	−0.42 (0.39)	ns	−0.29 (0.36)	ns
Dysphoria	−0.66 (0.22)	0.02	0.37 (0.20)	ns	−0.28 (0.16)	ns
Anxiety	−0.24 (0.19)	ns	0.18 (0.20)	ns	−0.10 (0.16)	ns
Euphoria	0.29 (0.36)	ns	0.09 (0.35)	ns^c^	0.37 (0.31)	ns
Apathy	−0.43 (0.17)	0.01	0.11 (0.17)	ns	−0.32 (0.15)	0.03
Disinhibition	−0.01 (0.25)	ns	−0.15 (0.25)	ns	−0.14 (0.21)	ns
Irritability	−0.29 (0.19)	ns	0.15 (0.19)	ns	−0.22 (0.15)	ns
Motor	−0.87 (0.39)	0.02	1.31 (0.40)	<0.01	0.08 (0.29)	ns
Sleep	−0.12 (0.20)	ns	−0.05 (0.20)	ns	−0.13 (0.17)	ns
Appetite	−0.35 (0.17)	0.04	−0.02 (0.17)	ns	−0.32 (0.15)	0.03
Total	−0.50 (0.17)	<0.01	0.13 (0.17)	ns	−0.37 (0.14)	<0.01

Abbreviations: AD, Alzheimer's disease; DLB, dementia with Lewy bodies; FTLDrs, frontotemporal lobar degeneration–related disorders; NPI, Neuropsychiatric Inventory; NPTX2, neuronal pentraxin 2; p‐tau, phosphorylated tau; SE, standard error.

^a^ Significant in AD.

^b^ Significant in DLB.

^c^ Significant in FTLDrs.

NPTX2/p‐tau associated with hallucinations in the DLB group (β = −0.89, SE = 0.48, *P *< 0.05; Table S3), and with dysphoria (β = −0.66, SE = 0.22, *P *< 0.05), apathy (β = −0.43, SE = 0.17, *P *< 0.05), motor (β = −0.87, SE = 0.39, *P *< 0.05), appetite (β = −0.35, SE = 0.17, *P *< 0.05), and the total NPI score in all individuals (β = −0.50, SE = 0.17, *P *< 0.01). P‐tau showed no significant NPI association other than with the motor subscore in all individuals (β *= *1.31, SE = 0.40, *P *< 0.01). Last, NPTX2 was associated with apathy (β = −0.32, SE = 0.15, *P *< 0.05) and total NPI scores (β = −0.37, SE = 0.14, *P *< 0.05) in all individuals. No significant associations were found between any marker and the FTLDrs group.

Overall, the biomarkers showed similar association with NPI, although NPTX2/p‐tau better reflected several subscores, particularly in DLB and for dysphoria, apathy, and the total NPI score overall. However, many differences between the markers were negligible.

### 3.4 Associations with gray matter atrophy

We analyzed the relationship between NPTX2 and structural brain changes in the participants with an MRI that passed quality control: FTLDrs (*n* = 73), AD (*n* = 68), DLB (*n* = 42), and CU (*n* = 66). In the full dataset (Figure S8 in supporting information), NPTX2 was associated with reduced gray matter volume in the temporal cortex, including the hippocampus, insula, medial prefrontal regions, and thalamus. Similar results were observed with NPTX2/p‐tau. P‐tau had a weaker association with gray matter volume. These associations were partially retrieved, to a lower extent, in the different diagnostic groups (data not shown).

## 4 DISCUSSION

In this study, the levels of CSF NPTX2 in patients with AD, DLB, and FTLDrs were analyzed using a novel in‐house Simoa NPTX2 assay. NPTX2/p‐tau was assessed across diagnostic groups, and cognitive and psychiatric measures, which were compared to NPTX2 and p‐tau as single markers. We found that NPTX2/p‐tau performed better than NPTX2 and p‐tau to distinguish healthy individuals from patients with DLB and FTLDrs, and better correlated with cognition and cognitive decline, as well as psychiatric features.

Our findings corroborate other studies reporting on lower CSF NPTX2 levels across neurodegenerative diseases,^9^, ^10^, ^16^ compared to CU individuals. We also demonstrated for the first time that NPTX2 has significantly lower levels in the collective FTLDrs group compared to AD and DLB. Moreover, NPTX2 proved to be a strong predictor of dementia, correlating with several cognitive and psychiatric measures. The decrease in NPTX2 is likely due to the synaptic dysfunction and loss prevalent in these syndromes,^6^, ^29^, ^30^ especially because decreases in NPTX2, a protein responsible for synaptic plasticity and signaling, have been associated with increased brain atrophy, which we also observed.^8^, ^31^ Unlike other synaptic proteins reported in CSF that change primarily with amyloid pathology and therefore may not fully capture synaptic damage,^32^ NPTX2 appears to reflect broader synaptic integrity. Therefore, our results reinforce its robustness and utility as a biomarker of synaptic health, especially in those with AD.^8^, ^9^, ^10^

In line with previous findings on the utility of synaptic protein ratios by Nilsson et al.,^9^ NPTX2/p‐tau had a vastly better diagnostic performance compared to NPTX2, as it differentiated all clinical groups from CU individuals to a greater extent than NPTX2. Overall, NPTX2/p‐tau also performed better than p‐tau to differentiate the non‐AD groups from the CU group, while p‐tau remains the most indicated biomarker to discriminate AD from CU individuals, as part of the AD core biomarkers.^33^ Both NPTX2 and NPTX2/p‐tau also performed better than p‐tau in discriminating between the FTLDrs and CU groups.

Furthermore, the relationship between NPTX2/p‐tau with cognition was notably stronger. Like NPTX2, NPTX2/p‐tau was strongly associated with MMSE in all individuals, to a greater extent than p‐tau. Additionally, NPTX2/p‐tau was associated with MMSE within the AD and DLB subgroups, while p‐tau only associated with MMSE within DLB, suggesting that, despite not improving discrimination of AD from CU, NPTX2/p‐tau might be more useful in reflecting cognitive changes, compared to NPTX2 or p‐tau.

The relationship between NPTX2/p‐tau and cognitive decline was stronger overall, compared to p‐tau or NPTX2. NPTX2/p‐tau was not only associated with longitudinal decline of MMSE, but its levels were also progressively decreased with increases in the CDR score and the GDS, the latter both cross‐sectionally and longitudinally, including longitudinally within the DLB group.

NPTX2/p‐tau was also the strongest predictor of dementia overall, and in AD and DLB individually. Accordingly, NPTX2/p‐tau was also more strongly associated with changes within the cognitive domains.

While episodic memory, being more affected in AD, was better explained by p‐tau, NPTX2/p‐tau demonstrated stronger association with attention/executive function, language, and visuospatial measures than either of the standalone biomarkers. These effects were also seen within the AD and DLB subgroups, and may be particularly useful in DLB, in which executive function and language are most affected.^34^, ^35^ Finally, these global cognitive changes were reflected by the extent of brain atrophy, which was highly associated with both NPTX2 and NPTX2/p‐tau, as well as p‐tau to a lesser degree.

Similar to NPTX2 and p‐tau, we did not observe a clear relationship between NPTX2/p‐tau and NPI. However, NPTX2/p‐tau did display a stronger association with neuropsychiatric scores than standalone markers, being associated with hallucinations in DLB, one of the most common psychiatric symptoms in the disease. This suggests that the levels of NPTX2, as well as its ratio with p‐tau, might be able to predict psychiatric‐related symptoms, especially because NPTX2 has been tentatively linked to neuropsychiatric symptoms and mood disorders.^36^, ^37^

The improvement observed with NPTX2/p‐tau over the individual biomarkers for both diagnostic group discrimination and association with cognitive measures reflects previous findings with other synaptic protein ratios (i.e., SNAP‐25/NPTX2 and 14‐3‐3 zeta/delta/NPTX2).^9^ Here, we not only emphasize the potential of ratios with synaptic proteins, but also expand on limited literature, which has been exclusively in the context of MCI and the AD continuum.^9^, ^20^

Our findings suggest that NPTX2/p‐tau offers advantages over p‐tau alone when assessing cognition, cognitive decline, and neuropsychiatric symptoms, particularly in DLB. This is notable because p‐tau is currently one of the best performing markers, yet our data show weaker associations compared to NPTX2/p‐tau. A similar pattern is observed in FTLDrs. This may be because p‐tau plays a smaller role in DLB and FTLDrs, resulting in NPTX2 contributing additional information when combined with p‐tau, leading to a better reflection of cognitive changes than either marker alone. However, p‐tau remains the strongest marker in AD, most likely due to its close relationship with the clinical phenotype of AD. Together with previous studies, our results support further investigation into ratios between synaptic proteins and their relationship with cognitive decline.

This study had several limitations. First, follow‐up samples were only available for 57% of participants, and later time points included fewer individuals, which may have reduced the statistical power in some longitudinal analyses. Additionally, a limited number of individuals within each FTLDrs subtype required us to analyze FTLDrs as a single group, possibly decreasing sensitivity to detect changes to specific diseases within the FTLDrs spectrum. Furthermore, given the known pathological heterogeneity of FTLDrs, the observed differences likely reflect a mixture of tau‐ and TAR DNA‐binding protein 43–related processes, rather than a single mechanism. Conversely, a key strength of our study is the large and well‐characterized SPIN cohort, which enabled comprehensive analyses of cognition and its longitudinal change, with follow‐up extending to almost 10 years. Furthermore, to our knowledge, this study includes one of the largest sets of patients with DLB and FTLDrs with CSF NPTX2 measures reported to date.

In this study, we used an in‐house Simoa assay to quantify CSF NPTX2 and evaluated its utility, both alone and as a ratio with p‐tau, to distinguish AD, DLB, and FTLDrs from CU, as well as to assess their relationship with cognitive and neuropsychiatric features. Overall, NPTX2/p‐tau outperformed both biomarkers alone, particularly in DLB and FTLDrs, and showed stronger association with cognition, cognitive decline, and neuropsychiatric symptoms. Together, this suggests that combining NPTX2 and p‐tau, which reflect different aspects of the neurodegenerative process, provides complementary information that is better captured when using the ratio, compared to the standalone markers. While additional work in pathology‐confirmed cohorts is needed, our results support consideration of using NPTX2 as a tool in evaluating cognitive decline, especially beyond AD.

## CONFLICT OF INTEREST STATEMENT

D.A. participated in advisory boards from Fujirebio‐Europe, Roche Diagnostics, Grifols SA, and Lilly, and received speaker honoraria from Fujirebio‐Europe, Roche Diagnostics, Nutricia, Krka Farmacéutica SL, Zambon SAU, and Esteve Pharmaceuticals SA. A.L. has served as a consultant or on advisory boards for Almirall, Beckman Coulter, Biogen, Fujirebio‐Europe, Roche, Grifols, Novartis, Eisai, Lilly, and Nutricia. O.B., D.A., A.L., and J.F. declare a filed patent application (WO2019175379 A1 Markers of synaptopathy in neurodegenerative diseases). H.Z. has served on scientific advisory boards and/or as a consultant for Abbvie, Acumen, Alector, Alzinova, ALZPath, Amylyx, Annexon, Apellis, Artery Therapeutics, AZTherapies, Cognito Therapeutics, CogRx, Denali, Eisai, LabCorp, Merry Life, Nervgen, Novo Nordisk, Optoceutics, Passage Bio, Pinteon Therapeutics, Prothena, Red Abbey Labs, reMYND, Roche, Samumed, Siemens Healthineers, Triplet Therapeutics, and Wave; has given lectures in symposia sponsored by Alzecure, Biogen, Cellectricon, Fujirebio, Lilly, Novo Nordisk, and Roche; and is a co‐founder of Brain Biomarker Solutions in Gothenburg AB (BBS), which is a part of the GU Ventures Incubator Program (outside submitted work). K.B. has served as a consultant and on advisory boards for Abbvie, AC Immune, ALZPath, AriBio, BioArctic, Biogen, Eisai, Lilly, Moleac Pte. Ltd, Neurimmune, Novartis, Ono Pharma, Prothena, Roche Diagnostics, and Siemens Healthineers; has served on data monitoring committees for Julius Clinical and Novartis; has given lectures, produced educational materials, and participated in educational programs for AC Immune, Biogen, Celdara Medical, Eisai, and Roche Diagnostics; and is a co‐founder of Brain Biomarker Solutions in Gothenburg AB (BBS), which is a part of the GU Ventures Incubator Program, outside the work presented in this paper. All other authors report no conflicts of interest. Author disclosures are available in the Supporting Information.

## CONSENT STATEMENT

This study was approved by the local ethics committee (Dnr IIBSP‐DOW‐2014‐30) in conformity with the Declaration of Helsinki. Informed consent was obtained from all participants.

## Supporting information




**Supporting Information**: dad270391‐sup‐0001‐SuppMat.docx

**Supporting Information**: dad270391‐sup‐0002‐ICMJE.pdf
