# Supplementary material for: Cerebrospinal fluid NPTX2/p‐tau ratio as a biomarker for cognitive decline in neurodegenerative diseases
Source: Alzheimers Dement (Amst). 2026 Jun 18;18(2):e70391. doi: 10.1002/dad2.70391 (PMC13279535; doi:10.1002/dad2.70391)
Supplement: Supplementary file 1 — Supporting Information: dad270391‐sup‐0001‐SuppMat.docx [file DAD2-18-e70391-s001.docx]

**Supplementary data**

**Supplementary Text 1 - Clinical Cohort**

Participants from the Sant Pau Initiative on Neurodegeneration (SPIN cohort) were evaluated at the Sant Pau Memory Unit (Barcelona, Spain) between November 2013 and April 2023. Full details of the SPIN cohort protocol are described in Alcolea et *al.* [1]. Participants in this cohort were patients with a diagnosis of AD (prodromal AD or AD dementia, classified according to NIA-AA guidelines, n=287) [2], DLB (probable DLB, n=118) [3], FTLDrs [probable bvFTD, n=55 [4], semantic variant primary progressive aphasia (svPPA), n=10 [5], nonfluent/agrammatic variant of primary progressive aphasia (nfvPPA), n=20 [5], corticobasal syndrome (CBS), and progressive supranuclear palsy (PSP), n=39 [6–8], total n=121], or cognitively unimpaired controls (CU, n=159), and are summarized in Table 1.

Inclusion criteria for controls required the absence of a cognitive or neurological disorder (MMSE 27–30, Clinical dementia rating = 0, FCSRT total immediate adjusted score > 7, absence of significant impairment in other domains or in daily living activities) and CSF AD biomarkers within the normal range. All participants received neurological and neuropsychological evaluation (Neuropsychiatric Inventory – NPI) and provided CSF samples. A fraction of participants (n=391, 57%) were followed for an average of 3.21 years (standard deviation (SD)=2.05), with some participants being followed up to 9.16 years. All participants were assessed for core AD biomarkers, namely brain amyloidosis (low CSF levels of Aβ_42_, CSF Aβ_42_/Aβ_40_ ratio or positive amyloid PET imaging), tau pathology and neurodegeneration (high CSF levels of p-tau and total tau (t-tau)) based on local cut-offs (CSF Aβ_42_ < 916 pg*/*mL, CSF Aβ_42_/Aβ_40_ < 0.062, CSF p-tau > 63 pg*/*mL, and CSF t-tau > 456 pg/mL). These cut-offs have high specificity and sensitivity to distinguish AD dementia patients from controls as previously described [9].

**Supplementary Text 2 - NPTX2 measurements**

The measurements of CSF NPTX2 were performed on a Simoa HD-X instrument (Quanterix) using the recently developed in-house assay, at the University of Gothenburg, Mölndal, Sweden [10]. Briefly, 10 µL of CSF were used to quantify NPTX2 using our in-house assay, which included a rabbit monoclonal antibody (clone: EPR15618, catalogue number: ab191563, Abcam) and a biotinylated rabbit monoclonal antibody (clone: EPR24020-38, catalogue number: ab277533, Abcam), as capture and detector antibodies, respectively. The full-length recombinant NPTX2 (7816-NP-050, Biotechne) was used as the calibrator, and the Tau 2.0 diluent (#101556 Quanterix) was used to dilute all samples and calibrators. Singlicate measurements of CSF NPTX2 were performed in 688 participant samples across 13 analytical runs. Internal quality controls samples were measured at the beginning and end of each analytical run to assess intra- and inter-plate variability. All clinical samples were above LOD and LLOQ of the assay (1.14 pg/mL and 2.70 pg/mL, respectively) and 1 (0.1%) sample had a technical failure and was excluded from the analysis, resulting in 687 participants in the study [10].

**Supplementary Text 3 - MRI acquisition and analysis**

A high-resolution three-dimensional T1-weighted image was acquired in a 3T Philips-Achieva scanner at Hospital del Mar (Barcelona, Spain) or a 3T Siemens Prisma scanner at Hospital Clinic (Barcelona, Spain). The detailed acquisition parameters of the imaging protocols are provided in Supplementary Table 1. We used the Computational Anatomy Toolbox (CAT12, https://neuro-jena.github.io/cat/) for the SPM12 software (Wellcome Centre for Human Neuroimaging, University College London, Queen Square Institute of Neurology) to preprocess the structural 3D-T1 sequence and extract the total intracranial volume. Segmented and modulated gray matter maps were smoothed using an 8-mm full-width at half-maximum Gaussian kernel for voxelwise analyses. Of the 257 participants with available MRI, eight were excluded due to suboptimal image quality as evaluated by a CAT12 IQR superior to 3.

**Supplementary Text 4 - Statistical analysis**

Statistical analysis was performed using the R environment (v4.4.0) [11]. Continuous variables in the cohort demographics were assessed using analysis of variance (ANOVA), while categorical variables were assessed by chi-square goodness-of-fit test. Group-wise comparisons for NPTX2, p-tau, and the NPTX2/p-tau ratio were performed using linear models after log-transformation to achieve normality, adjusted for multiple using the Benjamini–Hochberg false discovery rate (FDR), with p-values < 0.05 considered significant. Receiver operating characteristic (ROC) curves were calculated, resulting in an area under the curve (AUC), and the DeLong test was used to compare different AUCs. Associations with clinical features and disease progression were addressed using linear regression, linear-mixed-effects regression (random intercept and slopes), or cumulative logit mixed-effects regression (ordered logistic), after standardization of biomarker variables. Continuous cognitive outcomes were standardized and analysed with linear models (the Trail-Making Test A and B were log-transformed and sign-reversed before standardization), while ordered categorical outcomes (Neuropsychiatric Inventory domain scores, NPI total score, and Poppelreuter Total) were analysed with proportional-odds (ordered logistic) regression. Cox regression analysis was performed to evaluate association between the biomarkers and progression to dementia, from which the hazard ratio (HR) was generated (shown inverted). All analyses were adjusted for age, sex, and education as confounders.

For neuroimaging data, we performed voxelwise analyses on the complete dataset, adjusting for age, sex, years of education, and total intracranial volume. Due to the small size of each clinical group with available neuroimaging, analyses in each subgroup were not adjusted for covariates except TIV. Voxel-wise analyses were performed within a grey matter (GM) mask excluding non-grey matter voxels. Multiple comparisons were corrected using a family-wise error (FWE) threshold of *P*<0.05 and a minimum cluster size of 500 mm³. To detect more subtle effects, voxel-wise results were also presented at an uncorrected threshold of *P*<0.005 with a cluster size of k>500 mm³.

**Supplementary Table 1**. 3T T1-weighted acquisition protocols.

|  | 3T Philips – Achieva (Hospital del Mar) | 3T Siemens - Prisma fit  (Hospital Clinic) | |
| --- | --- | --- | --- |
|  | Protocol | Protocol 1 | Protocol 2 |
| **Echo Time (ms)** | 3.8 | 2.98 | 2.96 |
| **Repetition Time (ms)** | 8.2 | 2300 | 2300 |
| **Flip Angle** | 8 | 9 | 9 |
| **Number of Slices** | 160 | 240 | 208 |
| **Slice Thickness (mm)** | 1 | 1 | 1 |
| **Field of View (mm²)** | 256x256 | 256x256 | 256x256 |
| **Voxel size (mm)** | 0.94x0.94x1 | 1x1x1 | 1x1x1 |

**

**

**Supplementary Figure 1. Biomarker levels in CU vs. DLB and FTLDrs. (A)** NPTX2 in CU (n=159) and DLB (n=113), **(B)** p-tau in CU (n=159) and DLB (n=111), **(C)** NPTX2/p-tau in CU (n=159) and DLB (n=111), **(D)** NPTX2 in CU (n=159) and FTLDrs (n=120), **(E)** p-tau in CU (n=159) and FTLDrs (n=109), **(F)** NPTX2/p-tau in CU (n=159) and FTLDrs (n=109). P-values in boxplots were obtained from linear models adjusted for age, sex, and education. Horizontal line within boxplots denotes median, top and bottom of boxplots represent the 25th and 75th percentiles, respectively, and whiskers extend to 1.5x interquartile range. FC = fold change, CI = confidence interval.


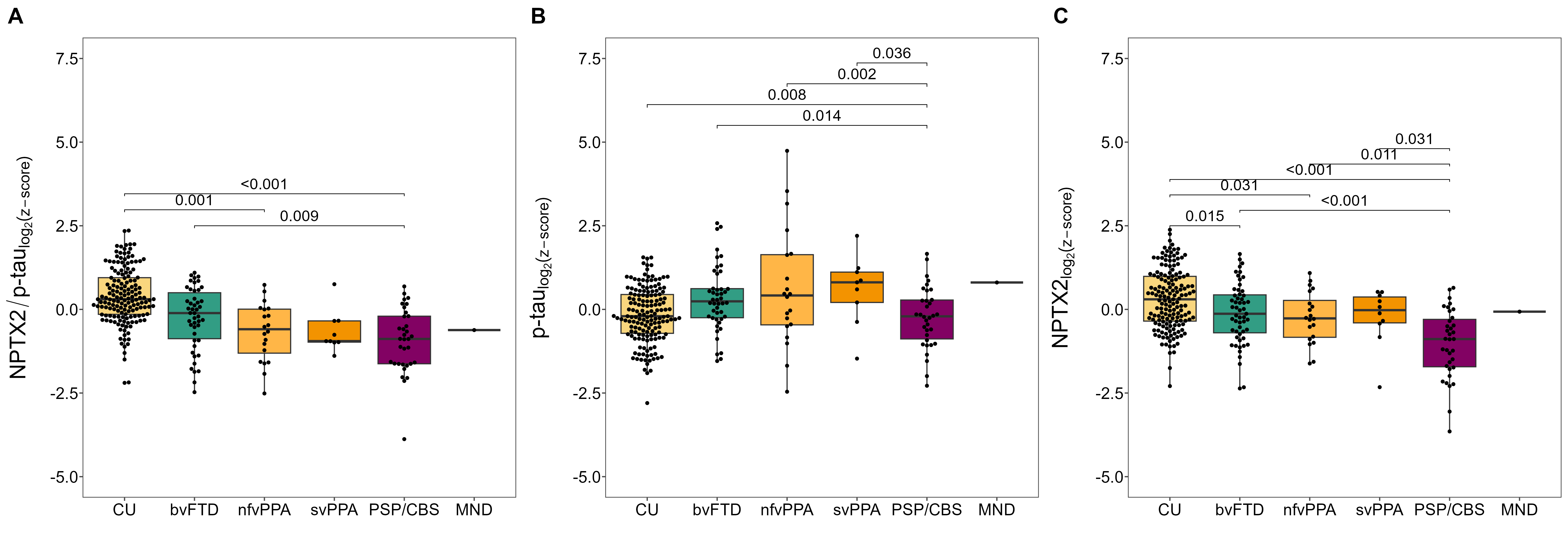


**Supplementary Figure 2. Biomarker levels in CU and FTLDrs subgroups. (A)** NPTX2/p-tau in CU (n=159), bvFTD (n=46), nfvPPA (n=20), svPPA (n=9), PSP/CBS (n=33) and MDN (n=1), **(B)** p-tau in CU (n=159), bvFTD (n=46), nfvPPA (n=9), svPPA (n=9), PSP/CBS (n=33) and MDN (n=1), **(C)** NPTX2 in CU (n=159), bvFTD (n=55), nfvPPA (n=20), svPPA (n=10), PSP/CBS (n=34) and MDN (n=1). P-values were obtained from a linear regression model with age, sex, and education as covariates. All biomarker levels were log-transformed to aid data visualization and results remained consistent when subgroups with n<10 were excluded prior analysis.

**Supplementary Table 2. Summary of group comparisons**. Standardized estimates and AUC values for the comparisons between diagnostic groups and the control group, as well as standardized estimates for comparisons between diagnostic groups.

|  | AD | DLB | FTLDrs | AD vs DLB | AD vs FTLDrs | DLB vs FTLDrs |
| --- | --- | --- | --- | --- | --- | --- |
|  | **β (SE)** | | | | | |
| NPTX2 | -0.34 (0.13) | -0.45 (0.16) | -0.72 (0.14) | -0.11 (0.11) | -0.38 (0.11) | -0.28 (0.13) |
| p-tau | 1.45 (0.09) | 0.47 (0.11) | -0.01 (0.10) | 0.99 (0.08) | 1.44 (0.08) | 0.46 (0.09) |
| NPTX2/p-tau | -1.43 (0.11) | -0.74 (0.13) | -0.59 (0.13) | -0.69 (0.09) | -0.86 (0.09) | -0.17 (0.11) |
|  | **AUC (95%CI)** | | | | | |
| NPTX2 | 0.59 (0.54-0.65) | 0.63 (0.56-0.69) | 0.70 (0.63-0.76) | - | - | - |
| p-tau | 0.99 (0.99-1) | 0.75 (0.69-0.81) | 0.59 (0.52-0.67) | - | - | - |
| NPTX2/p-tau | 0.95 (0.93-0.97) | 0.79 (0.74-0.85) | 0.78 (0.72-0.83) | - | - | - |


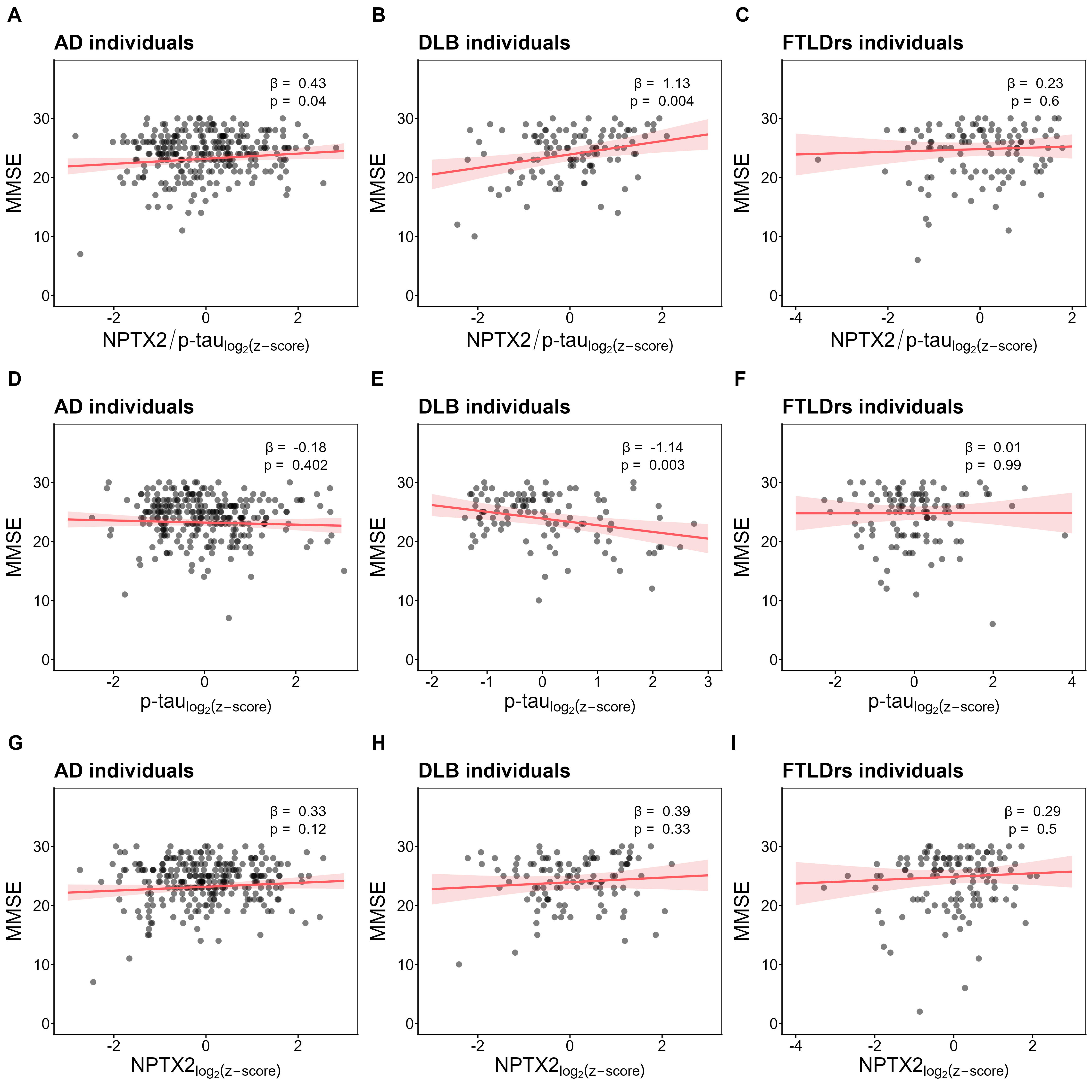


**Supplementary Figure 3. Association with MMSE scores across the different diagnostic groups.** Specifically, assoaciations of MMSE and **(A)** NPTX2/p-tau in AD (n=287), **(B)** NPTX2/p-tau in DLB (n=112), **(C)** NPTX2/p-tau in FTLDrs (n=110), **(D)** p-tau in AD (n=287), **(E)** p-tau in DLB (n=112), **(F)** p-tau in FTLDrs (n=110), **(G)** NPTX2 in AD (n=287), **(H)** NPTX2 in DLB (n=118), **(I)** NPTX2 in FTLDrs (n=121).





**Supplementary Figure 4. Cross-sectional and longitudinal p-tau association with MMSE, GDS, and CDR for all individuals. (A)** Association of p-tau with MMSE, at baseline (n=671). **(B)** Longitudinal trajectory of MMSE according to p-tau levels (1288 data points from 371 individuals followed-up for an average of 3.18 years (SD of 2.06)). **(C)** P-tau levels across the GDS, at baseline (n=671). **(D)** Longitudinal probability of a given GDS according to p-tau levels (tertiles) (1287 data points from 371 individuals followed-up for an average of 3.18 years (SD of 2.06)). **(E)** P-tau levels across global CDR in HC, AD, and DLB individuals (n=270), at baseline. **(F)** P-tau levels across FTLD-specific global CDR in FTLDrs individuals (n=24), at baseline. All analyses were obtained from linear regression, linear-mixed models (random slopes and intercept), or ordered logistic regression, with age, sex, and education as covariates and with false discovery correction performed for multiple comparisons in (C), (E) and (F). P-tau was log-transformed to aid data visualization and estimate interpretation. All estimates and p-values in (B) and (D) are compared to p-tau-Low.





**Supplementary Figure 5. Cross-sectional and longitudinal NPTX2 association with MMSE, GDS, and CDR for all individuals. (A)** Association of NPTX2 with MMSE, at baseline (n=674). **(B)** Longitudinal trajectory of MMSE according to NPTX2 levels (1332 data points from 385 individuals followed-up for an average of 3.21 years (SD of 2.06)). **(C)** NPTX2 levels across the GDS, at baseline (n=681). **(D)** Longitudinal probability of a given GDS according to NPTX2 levels (tertiles) (1231 data points from 371 individuals followed-up for an average of 3.21 years (SD of 2.06)). **(E)** NPTX2 levels across global CDR in HC, AD, and DLB individuals (n=270), at baseline. **(F)** NPTX2 levels across FTLD-specific global CDR in FTLD individuals (n=24), at baseline. All analyses were obtained from linear regression, linear-mixed models (random slopes and intercept), or ordered logistic regression, with age, sex, and education as covariates and with false discovery correction performed for multiple comparisons in (C), (E) and (F). NPTX2 was log-transformed to aid data visualization and estimate interpretation. All estimates and p-values in (B) and (D) are compared to NPTX2-High.


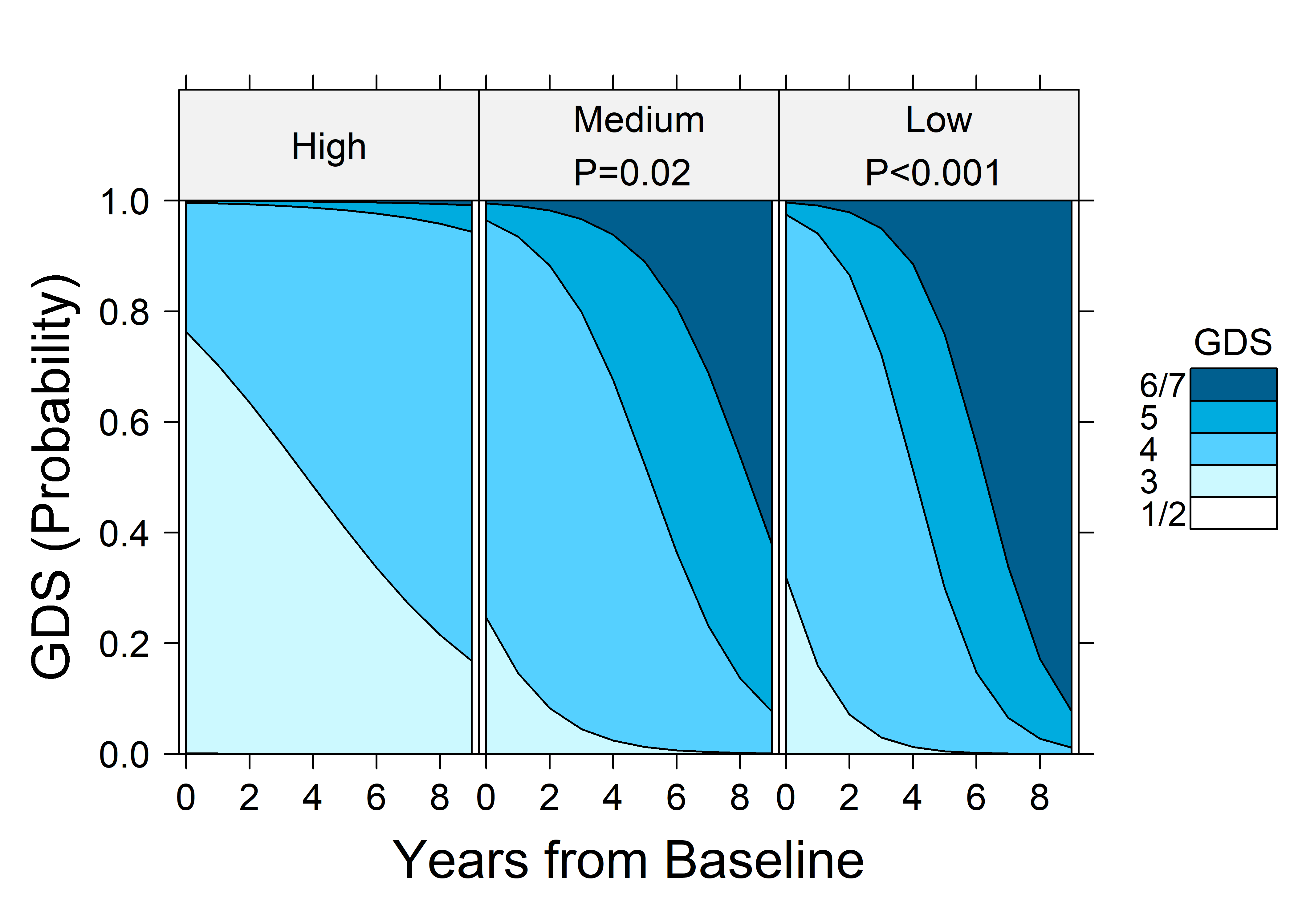


**Supplementary Figure 6. Longitudinal progression of GDS in DLB individuals.** Probability of a given GDS according to NPTX2/p-tau levels (tertiles), adjusted for age, sex, and education as covariates. The DLB group included 456 data points from 91 individuals followed-up for an average of 4.07 years (SD of 2.44)).





**Supplementary Figure 7. Kaplan-Meier curves depicting progression of AD, DLB, and FTLDrs individuals with normal cognition or MCI to dementia. (A)** NPTX2/p-tau in AD individuals (n=84), **(B)** NPTX2/p-tau in DLB individuals (n=46), **(C)** NPTX2/p-tau in FTLDrs individuals (n=25), **(D)** NPTX2 in AD individuals (n=85), **(E)** NPTX2 in DLB individuals (n=49), **(F)** NPTX2 in FTLDrs individuals (n=29). (**G**) p-tau in AD (n=84), (**H**) p-tau in DLB (n=46), (**I**) p-tau in FTLDrs (n=25). In the AD group, 70% of individuals converted to dementia with an average time to conversion of 2.91 years (SD of 1.54), in the DLB group, 69% of patients converted to dementia, with an average time to conversion of 2.58 years (SD of 2.09), and in the FTLDrs group, 72% of patients converted to dementia, with an average time to conversion of 2.76 years (SD of 1.46). Statistical models were performed with continuous variables, while tertiles were used for visualization purposes. P-value and curves reported in plots reflect Cox regression analysis adjusted for age, sex, and education.

**Supplementary Table 3. Significant associations between cognitive and neuropsychiatric measures in AD and DLB.**

|  |  |  | **NPTX2** | | **p-tau** | | **NPTX2/p-tau** | |
| --- | --- | --- | --- | --- | --- | --- | --- | --- |
|  | **Domain** | **Test/Subscore** | **β (SE)** | ***P*** | **β (SE)** | ***P*** | **β (SE)** | ***P*** |
| **AD** | Episodic memory | FCSRT | - | - | -0.23 (0.11) | 0.03 | 0.23 (0.12) | 0.05 |
|  | Attention/Execute function | TMT-A | - | - | - | - | 0.21 (0.10) | 0.04 |
|  | Language | Semantic fluency | 0.22 (0.10) | 0.03 | - | - | 0.27 (0.10) | 0.01 |
|  | Visuospatial skills | VOSP | - | - | -0.22 (0.11) | 0.04 | - | - |
| **DLB** | Attention/Execute function | Phonemic Fluency | - | - | - | - | 0.36 (0.15) | 0.02 |
|  |  | TMT-A | - | - | -0.37 (0.14) | 0.01 | 0.29 (0.15) | 0.06 |
|  | Language | Semantic fluency | - | - | - | - | 0.33 (0.16) | 0.05 |
|  | Visuospatial skills | Poppelreuter Total | - | - | -1.03 (0.33) | <0.01 | 1.05 (0.36) | <0.01 |
|  | NPI | Hallucinations | - | - | - | - | -0.89 (0.48) | 0.04 |

Abbreviations: FCSRT, Free and Cued Selective Reminding Test; TMT-A, Trail-Making test form A; VOSP, Visual Object Space and Perception; NPI, neuropsychiatric inventory.


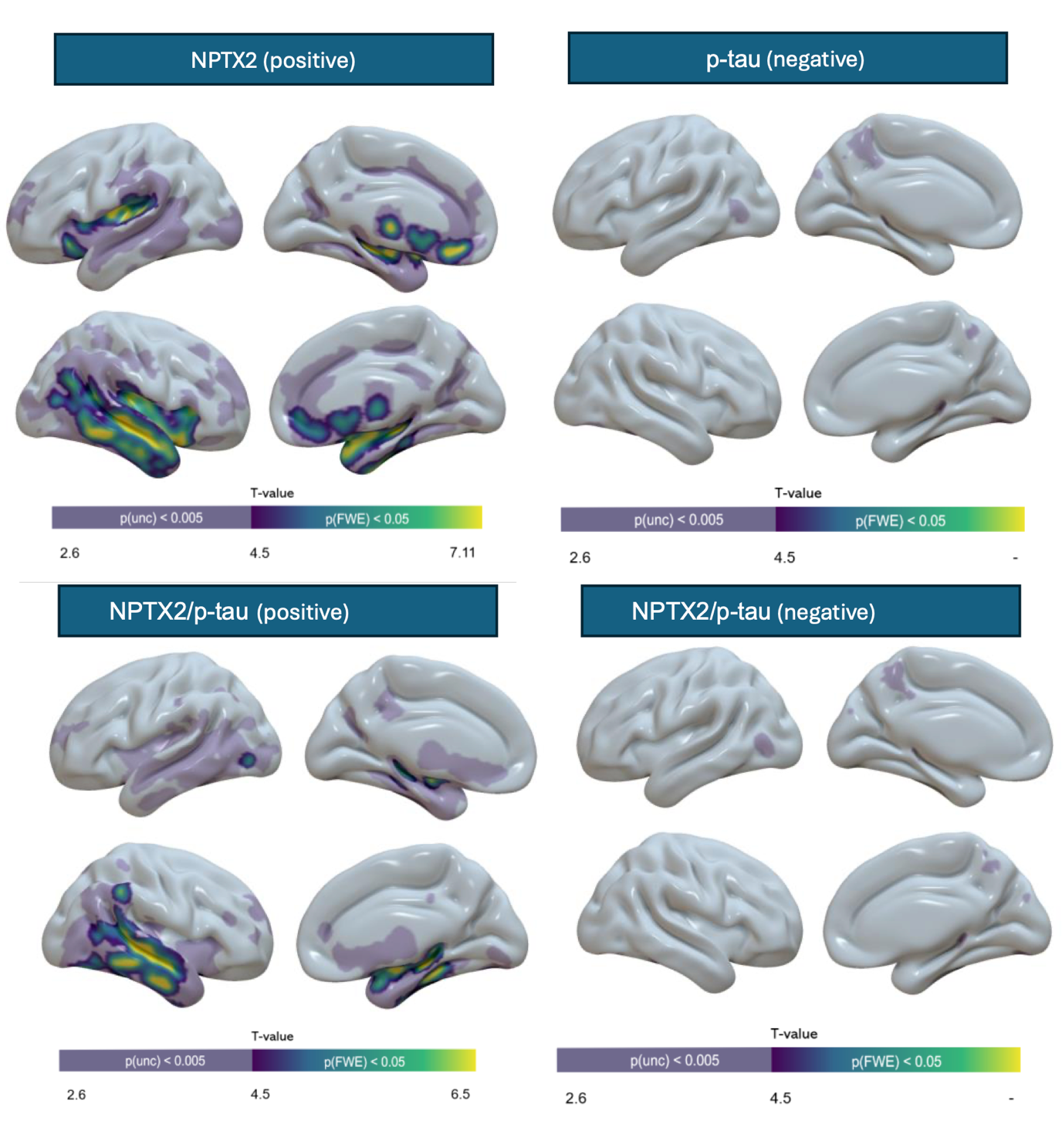


**Supplementary Figure 8.** **Association between cortical volume and the biomarkers.** Voxel-wise analyses were performed within a grey matter mask excluding non-grey matter voxels, in all individuals (n=257), adjusting for age, sex, years of education, and total intracranial volume.

**References**

1. Alcolea D, Clarimón J, Carmona-Iragui M, Illán-Gala I, Morenas-Rodríguez E, Barroeta I, et al. The Sant Pau Initiative on Neurodegeneration (SPIN) cohort: A data set for biomarker discovery and validation in neurodegenerative disorders. Alzheimers Dement (N Y). 2019 Oct 14;5:597–609.

2. Khachaturian ZS. Revised criteria for diagnosis of Alzheimer’s disease: National Institute on Aging-Alzheimer’s Association diagnostic guidelines for Alzheimer’s disease. Alzheimer’s & Dementia. 2011;7(3):253–6.

3. McKeith IG, Dickson DW, Lowe J, Emre M, O’Brien JT, Feldman H, et al. Diagnosis and management of dementia with Lewy bodies. Neurology. 2005 Dec 27;65(12):1863–72.

4. Rascovsky K, Hodges JR, Knopman D, Mendez MF, Kramer JH, Neuhaus J, et al. Sensitivity of revised diagnostic criteria for the behavioural variant of frontotemporal dementia. Brain. 2011 Sept;134(9):2456–77.

5. Gorno-Tempini ML, Hillis AE, Weintraub S, Kertesz A, Mendez M, Cappa SF, et al. Classification of primary progressive aphasia and its variants. Neurology. 2011 Mar 15;76(11):1006–14.

6. Armstrong MJ, Litvan I, Lang AE, Bak TH, Bhatia KP, Borroni B, et al. Criteria for the diagnosis of corticobasal degeneration. Neurology. 2013 Jan 29;80(5):496–503.

7. Boxer AL, Yu JT, Golbe LI, Litvan I, Lang AE, Höglinger GU. Advances in progressive supranuclear palsy: new diagnostic criteria, biomarkers, and therapeutic approaches. The Lancet Neurology. 2017 July 1;16(7):552–63.

8. Hoglinger GU, Respondek G, Stamelou M, Kurz C, Josephs KA, Lang AE, et al. Clinical Diagnosis of Progressive Supranuclear Palsy: The Movement Disorder Society Criteria. Mov Disord. 2017 June;32(6):853–64.

9. Alcolea D, Pegueroles J, Muñoz L, Camacho V, López‐Mora D, Fernández‐León A, et al. Agreement of amyloid PET and CSF biomarkers for Alzheimer’s disease on Lumipulse. Ann Clin Transl Neurol. 2019 Aug 28;6(9):1815–24.

10. Sauer M, Gomes BF, Shahrouki P, Lantero-Rodriguez J, Montoliu-Gaya L, Camporesi E, et al. Development and validation of a novel Simoa assay for NPTX2 in Alzheimer’s disease and Down syndrome. Alzheimer’s & Dementia. 2025;21(6):e70241.

11. R: The R Project for Statistical Computing [Internet]. [cited 2025 Nov 30]. Available from: https://www.r-project.org/
